# A measurement model for real estate bubble size based on the panel data analysis: An empirical case study

**DOI:** 10.1371/journal.pone.0173287

**Published:** 2017-03-08

**Authors:** Fengyun Liu, Deqiang Liu, Reza Malekian, Zhixiong Li, Deqing Wang

**Affiliations:** 1 China University of Mining & Technology, Xuzhou, Jiangsu Province, China; 2 Department of Economics, Kyoto University, Kyoto City, Kyoto Prefecture, Japan; 3 Department of Electrical, Electronic & Computer Engineering, University of Pretoria, Pretoria, Gauteng, South Africa; 4 Department of Manufacturing and Mechanical Engineering, UNSW Australia, Sydney, NSW, Australia; University of Rijeka, CROATIA

## Abstract

Employing the fundamental value of real estate determined by the economic fundamentals, a measurement model for real estate bubble size is established based on the panel data analysis. Using this model, real estate bubble sizes in various regions in Japan in the late 1980s and in recent China are examined. Two panel models for Japan provide results, which are consistent with the reality in the 1980s where a commercial land price bubble appeared in most area and was much larger than that of residential land. This provides evidence of the reliability of our model, overcoming the limit of existing literature with this method. The same models for housing prices in China at both the provincial and city levels show that contrary to the concern of serious housing price bubble in China, over-valuing in recent China is much smaller than that in 1980s Japan.

## 1. Introduction

Currently the Chinese economy attracts worldwide attention in order to maintain its high GDP growth rate after the global crisis in 2008. Meanwhile, the issue of a real estate bubble is a concern in recent years, and may cause the Chinese economy to go from “paradise” to “hell”. Therefore, it is very important to identify whether a bubble exists, and if it exists, to determine the extent. Thus, it is necessary to estimate the fundamental value of real estate, which is determined by economic fundamentals.

As Wang and Wang [1] emphasized, China is an ideal laboratory to study the influence of speculative demand versus fundamentals on property prices. In addition, Japan is a valuable reference to the recent situation in China since Japan experienced a real estate bubble in the 1980s. This paper estimates the fundamental value of real estate prices and the bubble sizes by prefecture in 1980s Japan as well as by province and city in recent China. Although previous studies have examined whether a bubble exists, an estimation of the bubble size by region and an empirical comparison of Japan and China are rare.

Kindleberger [2] defined a bubble as “a sharp rise in price of an asset or a range of assets in a continuous process, with the initial rise generating expectations of further rises and attracting new buyers–generally speculators, interested in profits from trading in the asset rather than its use or earning capacity”. This definition implies that, in a bubble, the price of the asset deviates from its “fundamental value”, and that a reversal of expectations and a sharp decline in prices (a crash) usually occur. Noguchi [3] defined a bubble as the part of land price that exceeds the theoretical land value, and found that Tokyo in 1987 had a land price bubble.

To identify a bubble, existing studies tend to examine the deviation of actual asset prices from their fundamental value. However, a consensus on a proper estimation method for the fundamental value of an asset has yet to be reached. Some define the fundamental property price as the discounted future imputed rents [4] or costs for owning a house [5]. The problem with this method is that there is no standard way to determine the future net revenue and the discount rate. Additionally, it is inappropriate in the case of China due to the lack of rental data. Some papers [5–7] use a partial equilibrium model to measure a bubble. However, the traditional method of using the housing value as the utility function of each period is not reasonable [4], and objective standards to select economic fundamental variables do not exist [8].

Another way is to use fundamental factors to estimate the asset fundamental value, and the difference between the fundamental value and the actual price is defined as a bubble. Recent studies have widely adopted this method [9–12]. This method allows the determinants for asset price to be easily understood and the bubble size to be examined. Although some countries lack data for rent and an efficient discount rate (e.g., China), this method is applicable. Existing literature is limited to whether a bubble exists, and only a few studies have tried to measure the bubble size of real estate in China [8]. Unlike previous studies, this paper proposes a method to estimate the housing price bubble size in China.

The selection of fundamental factors is crucial in this method because they may affect the residuals of the regression. To ensure accuracy, it is important to adopt a reference country that has experienced a bubble to test the reliability of the regression model. Because 1980s Japan is the most similar to recent China with regard to the bank-based financial system, financial liberalization reform, domestic currency appreciation, and soaring housing prices, it is used as the reference country. To the best of our knowledge, the previous literature has not adopted the case of 1980s Japan as a reference to test model reliability.

Because China is a large territory, the economic development level and characteristics vary by region. Quigley [13] asserted that heterogeneity exists in different regions of China; thus, it is important to consider the impact of local economic factors on housing prices. Obviously, a deeper study on housing markets could be attained with regional panel data rather than national data. Because commercial housing markets mainly exist in urban areas in China, city-level data is most appropriate. However, most previous studies involving regional analyses [14–16] have adopted provincial data. Although some [8, 17] employed data from China’s 35 major cities, they still ignored the influence of the local economy as the fundamental housing prices are evaluated as the future income (rental) flows. The current study uses panel local economic fundamental data from both China’s 35 major cities in 2001–2014 and 30 provinces/municipalities in 1994–2014. This paper reports original empirical analyses on the economic fundamentals and housing bubble size at both the city and provincial levels.

The remainder of the paper is as follows. Section 2 reviews related literature. Section 3 analyzes the theoretical measurement model for real estate bubble. Section 4 conducts empirical models and tests. Section 5 empirically examines bubble sizes of real estate in both 1980s Japan and recent China with panel data analysis. Section 6 outlines the conclusions.

## 2. Literature review

Although various studies have focused on China’s real estate bubble, a consensus on whether a bubble exists in China’s housing markets has not been reached. Following the time-varying income present value framework of Black et al. [18], Yu [8] using the panel VAR model and the data for China’s 35 major cities from the first quarter of 1999 to the second quarter of 2010 found that the housing bubbles in 35 cities are small, but eastern metropolises such as Beijing, Shanghai, and Shenzhen have experienced relatively big bubbles since 2005, which are irrational bubbles rather than rational bubbles. However, these results were challenged by Ren et al. [17], who followed the theory of rational expectation bubbles proposed by Blanchard and Watson [19], and employed discounting the future (rental) income flow as the fundamental house price. Based on panel data from 35 cities from 1999 to 2009, they did not find evidence of a real estate bubble in China. Obviously, the two papers draw inconsistent implications on the housing bubble issue, but both ignore the importance of local economic fundamentals.

Hui and Wang [11] examined the situation in Beijing and Shanghai with quarterly time series data from 1998 to 2012, and introduced the per capita disposable income of urban residents and floor space sold to represent the fundamentals on the demand side, and investment in commercial residential development and the lending rate as the supply side fundamental variables. They defined a bubble as a price anomaly when detecting an outlier in price residuals within the 90% confidence interval in vector error correction models (VECMs); only six and three anomalies were found in Beijing and Shanghai, respectively, suggesting that housing prices are reasonable most of the time. However, there is no standard or reliability test on the selection of variables in their papers.

Shih et al. [16] employed data from the first quarter of 2000 to the fourth quarter of 2012 in 28 Chinese provinces, a unit root and co-integration test on housing prices, and incomes. They found that most Chinese provinces have bubbles and affordability problems. A VECM, the Impulse Response function, and Granger causality found that the housing prices of north, east, and middle south regions are co-integrated. Housing prices in Beijing and Shanghai could affect those in peripheral (neighboring) provinces as an exogenous variable. The inter-provincial spillover in different regions is an advanced research point, while it seems to be a little slipshod to judge a non-stationary housing price series as a bubble.

With respect to the bubble size, Hui and Yue [10] conducted a typical study in which the interactions between housing prices and market fundamentals in Hong Kong, Beijing, and Shanghai were evaluated based on a comparative study. Because Hong Kong experienced a housing bubble in 1997, the test on its case could confirm the reliability of the method adopted. The stock of vacant new dwellings, disposable income of urban households, local GDP, and Shanghai stock price index were adopted as representatives of the economic fundamentals. PP tests, JJ tests, Granger causality tests, and Impulse Response functions were conducted. Based on the definition of a bubble as the difference between the actual measured housing prices and predicted housing prices, they found that around 22% of the housing price in 2003 was attributed to the bubble term in Shanghai, but Beijing did not have a bubble. However, the actual real estate prices typically fluctuate around their estimated fundamental value due to the error term of regression. Hui and Yue [10] did not exclude this rational fluctuation from the bubble size, but this research does.

Since the real estate market has regional features, this paper uses the same consideration as Hui and Yue [10] for the influence of local economies. Referencing the above limitations of existing literature, this study is an improvement from three aspects. First, we use the case of 1980s Japan to test the reliability of our model to overcome the lack of standardized selection criteria for fundamental variables. Second, we adopt panel data from China’s 35 major cities and 30 provinces/municipalities, and Japan’s 47 prefectures to compare the bubble sizes in the two countries at the city and regional levels. Third, the rational fluctuations of real estate prices around their fundamental value are removed from the bubble size.

## 3. Theoretical measurement model for real estate bubble size

To identify a bubble in real estate prices, we started with a macro model to depict the mechanism of price determination in the real estate market. Quigley [13], Hui and Yue [10], and Hui and Wang [11] assert that the real estate price is determined by the supply and demand in a comparative real estate market. That is,

$$P=H^{D},H^{S}$$ (1)

where P is the real estate price, H^D^ and H^S^ denote arrays of key factors of real estate demand and supply, respectively. According to economic theory, real estate demand is a function of real estate price and economic fundamentals—nominal GDP (NGDP) and a set of other exogenous variables X such as population density (PD) and the lending interest rate (LI). According to Jensen (1991) [20] and Thakur (2008, 2011) [21–22], economic fundamentals indicate the predictable economic size in a certain region, which can be tied to a number of key variables, such as gross product, population, and total gross output. Here we employ NGDP as the key variable, because it not only directly explains the economic size and economic development level, but also contains the inflation of prices. NGDP is the most representative variable of economic fundamentals. The increase in NGDP should lead to a rise in income and thus, increase the real estate demand. Since real estate is an asset as well as a consumable good, the growth of NGDP should cause inflation, stimulating people to invest in real estate for hedging and eventually elevating the demand in the real estate market. The rise of PD suggests population growth in the area, which requires more houses and consequently increases the real estate demand. The increase in LI enlarges costs of consumers when buying a house by banking loans, and thus decrease housing demand and prices. The demand function for real estate can be expressed as

$$H^{D}=d\left(P,NGDP,PD,LI\ldots \right)$$ (2)

Similarly, the real estate supply should be captured by a function of the real estate price and economic fundamentals—NGDP and a vector of exogenous variables Y such as the lending interest rate (LI). The growth of NGDP implies a rise in the output on a unit land, which increases the opportunity costs of land and consequently, decreases the supply due to real estate development enterprises. Similarly, the rise in the lending rate elevates the financing cost of real estate development enterprise, reducing the supply of real estate. The supply function for real estate can be expressed as

$$H^{S}=s\left(P,NGDP,LI,\ldots \right)$$ (3)

Substituting Eqs (2) and (3) into Eq (1) and solving for real estate prices, a reduced form of real estate price is obtained as Eq (4).

P=f(NGDP,PD,LI,…) (4)

Then, logarithmic forms of real estate prices (LRP), NGDP (LNGDP), and PD (LPD), and original LI are used to conduct a panel regression of real estate prices with an individual fixed effect as expressed as Eq (5), where i is the number of a certain region among the total of N regions, and t is a certain period among the total of T periods.

$$LRP_{it}=\text{c}+\alpha LNGDP_{it}+\beta LPD_{it}+\gamma LI_{it}+\delta_{i}D_{i}+u_{it},i=1,2,\ldots ,N;t=1,2,\ldots ,T$$ (5)

D_i_ is an individual dummy. Its value is 1 if i belongs to the individual i, otherwise it is 0. It is same in other formulations.

Following the viewpoint of Quigley [13] where the theoretical equilibrium price of real estate is determined by local economic fundamentals, Hui and Yue [10] estimated the real estate bubble as the difference between the actual real estate price and the theoretical fundamental price. Hui and Wang [11] point out that the discrepancy between the actual price and the theoretical one as a bubble is inaccurate because it contains the random disturbance term, which should appear in every regression of real estate prices by independent variables, including economic fundamentals.

Following Hui and Wang [11], we define the residual u_it_ = e_it_ + π_it_, where e_it_ is the rational fluctuation of real estate prices that include the random disturbance term, while π_it_ denotes abnormal movement, which is defined as a price anomaly, that is, a bubble or underpricing. The boundary (b) of e_it_ is defined as b = S.E. of regression > 0. If u_it_ is inside of [−b, b], then it fluctuates in a rational range and a real estate price bubble does not exist; if u_it_ is outside of [−b, b], then it is an anomaly, and the real estate price of i region in t period is overvalued (u_it_ > b) or undervalued (u_it_ < −b). Given LRP is a logarithm form of the actual real estate price, we used LRP* as the estimated real estate price in logarithmic form

$${\text{u}}_{it}=LRP_{it}{\;\text{-}\;\text{LRP}}_{it}^{*}=e_{it}+\pi_{it}$$ (6)

The estimated real estate price in the paper is the reasonable price in theory which is determined by the current economic fundamentals. The reliability of our model by employing the estimated real estate price is proved by the subsequent empirical study results which is consistent with the experience of 1980s Japan.

In theory, the bubble size (BS) is defined as Eq (7), where P is the actual real estate price, and P* is the estimated real estate price.

$$BS=\frac{P\text{-}P^{*}}{P^{*}}\times 100\%$$ (7)

Substituting Eqs (5) and (6) into Eq (7) allows the bubble size to be expressed as

$$BS=\frac{P\text{-}P^{*}}{P^{*}}\times 100\%=\frac{e^{LRP}\text{-}e^{LRP^{*}}}{e^{LRP^{*}}}\times 100\%=(e^{\left(LRP-LRP^{*}\right)}\text{-}1)\times 100\%=(e^{u}\text{-}1)\times 100$$ (8)

where if *u*_*it*_ = *e*_*it*_ + *π*_*it*_ ∈ [−*b*,*b*], then *π*_*it*_ = 0, implying no bubble. If *u*_*it*_ = *e*_*it*_ + *π*_*it*_ < −*b*, then *π*_*it*_ < 0, suggesting underpricing of real estate, and if *u*_*it*_ = *e*_*it*_ + *π*_*it*_ > *b*, then *π*_*it*_ > 0, implying overpricing and that a bubble may exist.

## 4. Empirical test

### 4.1. Data and modeling

This study adopts panel data from 47 prefectures between 1980 and 1999 for Japan. The average commercial land price (CP) and the average residential land price (RP) and NGDP in each prefecture are employed to represent the commercial and residential real estate price levels and the local economic fundamentals, respectively. To promote the accuracy of our tests, additional fundamental variables, PD (Population/Administer Area) and LI, which are from the demand and supply side, are introduced. The logarithmic forms of CP, RP, NGDP, and PD − LCP, LRP, LNGDP, LPD, and LI are entered into a panel regression model with individual-fixed effects. All the data are from Japan Bureau of Statistics (URL: http://www.stat.go.jp/).

Based on Eq (5), models for LCP and LRP are established as described by Eqs (9) and (10), respectively. When only introducing LNGDP to represent the fundamental economic variable, the model is called basic Models (1) and (3) for LCP and LRP, respectively. When LPD and LI are also introduced, these are called additional Model (2) and (4) for LCP and LRP, respectively.

$$LCP_{it}=\text{c}+\alpha LNGDP_{it}+\beta LPD_{it}+\gamma LI_{it}+\delta_{47}D_{47}+u_{it},i=1,2,\ldots ,47;\text{t}=1980,1981,\ldots ,1999$$ (9)

$$LRP_{it}=\text{c}+\alpha LNGDP_{it}+\beta LPD_{it}+\gamma LI_{it}+\delta_{47}D_{47}+u_{it},i=1,2,\ldots ,47;t=1980,1981,\ldots ,1999$$ (10)

For China, the panel data of 30 provinces/municipalities (Tibet is excluded from the 31 provinces/municipalities due to its unstable data) from 1994 to 2014 and 35 major cities from 2001 to 2014 are employed. The average residential commodity building prices (RCP) and average commercial commodity building prices (CCP) of 35 major cities are introduced to study the residential and commercial real estate bubbles, respectively. Because the data on residential and commercial housing prices in 1990s are unavailable, average commodity building prices (CBP) of 30 provinces/municipalities are employed to represent the overall average housing price level from 1994 to 2014. According to the National Bureau of Statistics of the People’s Republic of China, commodity buildings include residential buildings, villas and apartments, office buildings, commercial buildings, and others. Similarly, NGDP is the proxy of the local economic fundamentals. Additional fundamental variables (PD and LI) are employed. Their logarithmic forms (LCBP, LRCP, LCCP, LNGDP, LPD) and LI are entered into the panel regression model with individual fixed effects. The data source is the CEIC database and China Statistical Yearbook of various years between 1995 and 2015.

Eq (11) shows the model for the provincial level of LCBP. Eqs (12) and (13) show city levels of LRCP and LCCP, respectively. Similar to the tests for Japan, basic Model (5) only has LNGDP as the independent variable, while additional Model (6) also considers LPD and LI for commodity building prices in China’s 30 provinces/municipalities. Basic Model (7) and additional Model (8) are used for LRCP, and basic Model (9) and additional Model (10) are used for LCCP in China’s 35 major cities.

$$LCBP_{it}=\text{c}+\alpha LNGDP_{it}+\beta LPD_{it}+\gamma LI_{it}+\delta_{30}D_{30}+u_{it},i=1,2,\ldots ,30;t=1994,1995,\ldots ,2014$$ (11)

$$LRCP_{it}=\text{c}+\alpha LNGDP_{it}+\beta LPD_{it}+\gamma LI_{it}+\delta_{35}D_{35}+u_{it},i=1,2,\ldots ,35;t=1994,1995,\ldots ,2014$$ (12)

$$LCCP_{it}=\text{c}+\alpha LNGDP_{it}+\beta LPD_{it}+\gamma LI_{it}+\delta_{35}D_{35}+u_{it},i=1,2,\ldots ,35;t=1994,1995,\ldots ,2014$$ (13)

### 4.2. Test results

The above models are estimated using Eviews 6.0. Table 1 summarizes the estimation results of the panel regression for commercial and residential land prices in Japan.

**Table 1:** Estimation results in Japan (1980–1999).

Variables	Model(1)	Model(2)	Model(3)	Model(4)
	LCP	LCP	LRP	LRP
**LNGDP**	1.212***	1.737***	0.732***	0.734***
	(26.839)	-31.727	-29.405	-21.983
**LPD**		1.712***		2.692***
		-4.376		-11.285
**LI**		0.119***		0.037***
		-21.917		-11.075
**(C +C**_**i**_**)D**_**i**_^**1**^	yes	yes	yes	yes
**R-square**	0.847	0.932	0.901	0.947

Notes: Here only the key coefficients among the estimated results are summarized.

“***” represents the 1% level of significance. Numbers in parentheses are t-statistics.

The coefficients of LNGDP to LCP and LRP are 1.21 and 0.73, respectively, in basic models (1) and (3), indicating that a 1% increase of LNGDP causes a 1.21% growth in commercial land price and a 0.73% increase in residential land price. These results suggest that GDP is a significant factor for the increase of land price, but its influence on commercial land price is much larger than that on residential price. When introducing additional fundamental variables (LPD and LI), the coefficients of LNGDP, LDP, and LI to LCP are 1.74, 1.71, and 0.12, respectively, while those to LRP are 0.73, 2.70, and 0.04, respectively. These three variables have positive effects on land prices, which is consistent with the demand and supply theory. That is, growth of LNGDP increases local people’s income, the rise of LPD increases the demand for houses, and growth of LI increases the financing cost of real estate development. All of these decrease the supply, promoting an increase, which also increases real estate demand. The effects of LNGDP and LPD are strong, while those of LI are much smaller. The influence of LNGDP and LI to LCP are larger than those to LRP, whereas the impact of LPD to LCP is smaller. Apparently in 1980s Japan, the economic development level and the interest rate have larger effects on commercial land prices, while the population factor has a stronger influence on the residential land prices.

Similarly, panel regression basic Model (5) and additional Model (6) of the 30 provinces/municipalities, and basic Models (7) and (9), and additional Models (8) and (10) of 35 major cities in China are estimated (see Table 2).

**Table 2:** Estimation results in China.

Variables	30 Provinces/Municipalities (1994–2014)		35 Major Cities(2001–2014)			
	Model (5)	Model (6)	Model (7)	Model (8)	Model (9)	Model (10)
	LCBP	LCBP	LRCP	LRCP	LCCP	LCCP
**LNGDP**	0.628***	0.665***	0.726***	0.717***	0.652***	0.655***
	(87.209)	-66.029	-66.476	-60.852	-40.983	-38.105
**LPD**		-0.044		0.131***		0.154***
		(-0.472)		-2.893		-2.331
**LI**		0.025***		0.009		-0.032
		-6.705		(-0.690)		(-1.614)
**(C +C**_**i**_**)D**_**i**_^**1**^	yes	yes	yes	yes	yes	yes
**R-square**	0.951	0.955	0.944	0.945	0.854	0.857

Notes: Here only the key coefficients among the estimated results are summarized.

“***” represents the 1% level of significance. Numbers in parentheses are t-statistics.

The coefficients of LNGDP to LCBP are 0.63 and 0.67 in Models (5) and (6), respectively, at the provincial level, but are 0.73 and 0.72 to LRCP in Models (7) and (8), and 0.65 and 0.66 to LCCP in Models (9) and (10), respectively, at the city level. These results suggest that local economic growth has strong positive influence on housing prices. The coefficients of LNGDP to LRCP are larger than those to LCCP at the city level, showing that movements of economic fundamentals lead to larger fluctuations in residential housing prices rather than commercial housing prices. The coefficient of LPD to LCBP is not significant at the 90% confidence level in model (6), but is in model (8) and (10), implying that the population cannot interpret the housing price at the provincial level due to large rural areas, but it has a positive effect on the housing price at the city level. Although the impact of LI in model (6) is significant, it is very small (0.03) and is not significant at the 90% confidence level in model (8) and (10) due to the nonmarket-oriented interest rate system.

Interestingly, the coefficients of LNGDP to LRCB in 35 major cities in China (0.72–0.73) are very close to that of LNGDP to LRP in Japan at the prefectural level (0.73), indicating the reliability of our models for the two countries. Besides, the coefficients of LNGDP are significantly positive in both China and 1980s Japan, showing that local economic development should lead to growth in real estate price, and a 1% growth of the nominal GDP should realize an approximately 0.73% increase in residential real estate prices in China and Japan. The impact of population is much larger in Japan than that in China due to the much higher urbanization level in 1980s Japan.

## 5. Real estate bubble size measurement

### 5.1. Estimation of bubble sizes in 1980s’ Japan

Based on the regression results of Model (1), the commercial land price bubble size of Japan’s 47 prefectures is estimated according to Eq (8) (Tables 3–5). Here, the rational fluctuation of real estate price (e_it_) is removed using Eq (5), and only the abnormal movement (π_it_) is considered as a bubble. According to Table 3, in the late 1980s, most of Japan (30 prefectures) experienced land price bubbles. Commercial land prices in Kanto, Kinki, and Chubu areas had extremely large bubbles. In the Kanto area, Tokyo’s bubble peaked at 98.5% in 1987, followed by that of Chiba (82.0% in 1990), Kanagawa (53.5% in 1989), and Saitama (35.2% in 1991). The Kinki area had the largest bubbles in 1990 at 133.2% in Osaka, 94.7% in Kyoto, 77.3% in Nara, 66.2% in Hyogo, and 45.7% in Shiga. In the Chubu area, Aichi had the largest bubble (87.7%), followed by Shizuoka (31.8%) in 1990. Kyushu and Tohoku areas had medium bubbles, which peaked at 47.2% in 1991 and 47.0% in 1990, respectively. Chugoku, Hokkaido, and Shikoku areas experienced small bubbles, peaking at 28.1% in 1991, 15.9% in 1990, and 10.3% in 1990, respectively.

**Table 3:** Estimated commercial land price bubble sizes of Japan’s prefectures (%).

	Hokkaido	Aomori	Iwate	Miyagi	Akita	Yamagata	Fukushima	Ibaraki	Tochigi	Gumma	Saitama	Chiba	Tokyo	Kanagawa	Niigata	Toyama
**1980**	-	-	-	-22.2	-	-	-	-	-	-	-	-0.1	-30.4	-20.6	-	-
**1981**	-	-	-	-18.5	-	-	-	-	-	-	-	-2.9	-30.1	-19.2	-	-
**1982**	-	4.8	-	-5.1	0.3	-	-	-	-	-	-	-	-21.5	-14.6	-	-
**1983**	-	-	5.2	-	14.9	-	-	-	-	-	-	-	-6.1	-1.9	-	-
**1984**	-	10.3	-	-	11.6	-	-	-	-	-	-	-	-	-	-	-
**1985**	-	3.5	-	-	8.0	-	-	-	-	-	-	-	-	-	-	-
**1986**	-	-	-	-	-	-	-	-	-	-	-	-5.2	40.2	-	-	-
**1987**	-	-	-	-	-	-	-	-	-	-	-	10.9	98.5	22.1	-	-
**1988**	-	-	-	0.2	-	-	-	-	-	-	35.2	49.9	83.8	52.2	-	-
**1989**	-	-	-	26.8	-	-	-	-	-	-	34.1	67.5	64.7	53.5	-	-
**1990**	15.9	-	-	47.0	-	-	-	-	-	4.0	35.1	82.0	58.2	42.9	7.3	-
**1991**	13.8	-	-	41.0	-	-	-	-	13.1	12.1	35.2	69.8	54.8	38.1	10.8	4.7
**1992**	-	-	-	28.1	-	-	-	-	6.4	15.5	17.6	37.0	35.4	32.1	7.4	2.9
**1993**	-	-	-	10.9	-	-	-	-	-	-	-	-	-	-	-	-
**1994**	-	-	-	-	-	-	-	-	-	-	-	-	-	-	-	-
**1995**	-	-	-	-	-	-	-	-	-	-	-	-	-0.3	-	-	-
**1996**	-	-	-	-	-2.9	-	-	-	-	-	-1.0	-7.0	-22.3	-	-	-
**1997**	-0.1	-	-	-	-4.6	-	-	-	-	-	-16.0	-24.1	-32.1	-7.0	-	-
**1998**	-9.9	-7.4	-	-10.5	-8.0	-	-4.8	-0.7	-	-	-26.9	-33.8	-34.5	-13.3	-3.7	-1.5
**1999**	-25.5	-14.4	-	-17.5	-10.4	-	-17.4	-7.7	-1.2	-8.6	-35.5	-44.4	-38.9	-21.3	-14.0	-8.1

**Table 4:** Estimated commercial land price bubble sizes of Japan’s prefectures (%).

	Ishikawa	Fukui	Yamanashi	Nagano	Gifu	Shizuoka	Aichi	Mie	Shiga	Kyoto	Osaka	Hyogo	Nara	Wakayama	Tottori	Shimane
**1980**	-	-	-	-	-	-	-28.0	-	-	-23.4	-38.8	-	-	-	-	-
**1981**	-	-	-	-	-	-	-27.7	-	-	-5.4	-34.7	-	-	-	-	-
**1982**	-	-	-	-	-	-	-12.9	-	-	-	-	-	-	-	-	-
**1983**	-	-	-	-	-	-	-	-	-	-	-	-	-	5.1	-	17.7
**1984**	-	-	-	-	-	-	-	-	-	-	-	-	-	-	-	0.8
**1985**	-	-	-	-	-	-	-	-	-	-	-	-	-	-	-	1.0
**1986**	-	-	-	-	-	-	-	-	-	-	-	-	-	-	-	-
**1987**	-	-	-	-	-	-	-	-	-	-	26.2	-	-	-	-	-
**1988**	-	-	-	-	-	-	33.3	-	-	11.5	60.1	-	-	-	-	-
**1989**	-	-	-	-	-	10.5	61.3	-	-	54.4	111.4	35.6	29.2	-	-	-
**1990**	-	-	14.0	-	-	31.8	87.7	-	45.7	94.7	133.2	66.2	77.3	20.3	-	-
**1991**	3.9	-	19.5	-	4.4	30.4	72.8	4.0	18.7	61.0	104.4	42.1	72.2	11.7	-	-
**1992**	3.6	-	-	-	-	19.7	43.7	-	3.2	11.1	50.6	7.4	43.7	-	-	-
**1993**	-	-	-	-	-	-	8.5	-	-	-	1.1	-	-	-	-	-
**1994**	-	-	-	-	-	-	-	-	-	-	-	-	-	-	-	-
**1995**	-	-	-	-	-	-	-	-	-	-	-	-0.4	-	-	-	-
**1996**	-	-	-	-	-	-	-1.1	-	-	-5.4	-16.5	-14.3	-5.0	-0.8	-	-
**1997**	-	-	-6.1	-	-0.3	-	-9.5	-	-3.8	-11.5	-24.1	-14.6	-11.9	-6.3	-	-
**1998**	-	-	-14.5	-	-9.7	-8.4	-19.3	-	-5.4	-17.3	-29.5	-14.9	-14.6	-13.6	-	-
**1999**	-12.9	-6.0	-24.1	-7.8	-18.9	-19.3	-29.3	-	-12.3	-29.0	-39.0	-20.6	-21.6	-21.4	-	-

**Table 5:** Estimated commercial land price bubble sizes of Japan’s 45 prefectures (%).

	Okayama	Hiroshima	Yamaguchi	Tokushima	Kagawa	Ehime	Kochi	Fukuoka	Saga	Nagasaki	Kumamoto	Oita	Miyazaki	Kagoshima	Okinawa
**1980**	-	-4.6	-	4.4	-	-	-	-34.9	-	-	-	-	-	-	-3.5
**1981**	-	-5.0	-	10.7	-	-	-	-25.3	-	-	-	-	-	-	-
**1982**	-	-	-	15.0	-	-	-	-13.4	-	-	-	-	-	-	-
**1983**	-	-	-	12.9	-	-	-	-	-	-	-	-	-	-	-
**1984**	-	-	-	5.7	-	-	-	-	-	-	-	0.4	2.0	-	-
**1985**	-	-	-	6.6	-	-	-	-	-	-	-	2.5	-	-	-
**1986**	-4.3	-	-	-	-	-	-	-	-	-	-41.8	-	-	-	-
**1987**	-	-	-	-	-	-	-	-	-	-	-24.2	-	-	-	-
**1988**	-	-	-	-4.1	-	-	-	2.6	-	-	-	-	-	-	-
**1989**	-	-	-	-	-	-	-	12.6	-	-	9.1	-	-	-	-
**1990**	10.2	17.2	-	-	-	3.1	-	25.2	-	-	21.4	-	-	-	-
**1991**	28.1	17.5	-	-	10.3	7.9	-	31.3	-	7.0	26.5	-	-	-	47.2
**1992**	16.8	5.5	-	-	2.9	-	-	22.9	-	5.2	19.0	-	-	-	29.8
**1993**	-	-	-	-	-	-	-	2.9	-	-	6.8	-	-	-	-
**1994**	-	-	-	-	-	-	-	-	-	-	-	-	-	-	-
**1995**	-	-	-	-	-	-	-	-	-	-	-	-	-	-	-
**1996**	-	-	-	-	-	-	-	-	-	-	-	-	-	-	-
**1997**	-	-	-	-1.4	-0.4	-0.7	-	-	-	-	-	-	-	-	-
**1998**	-2.9	-	-	-4.5	-9.8	-7.9	-	-	-	-	-	-1.9	-	-3.7	-
**1999**	-11.5	-3.0	-	-8.6	-12.8	-12.1	-	-5.7	-	-	-0.8	-7.0	-10.0	-10.5	-3.6

By adopting the same procedure as Tables 3–5, the bubble size of the residential land price in Japan’s 47 prefectures is estimated (Tables 6–8). In 1980s Japan, 18 prefectures experienced land price bubbles; they seem to be geographically clustered in three areas: Tokyo-centralized, Osaka-centralized, and Aichi-centralized areas. In the Tokyo-centralized area, Tokyo is the largest, peaking at 80.4% in 1987, followed by Chiba (54.0% in 1990) and its surrounding prefectures. The Osaka-centralized area also had a large bubble, which peaked at 95.4% in Osaka, while Kyoto, Nara, Hyogo, and Shiga peaked at 85.6%, 73.0%, 44.1%, and 38.4%, respectively, in 1990. The Aichi-centralized area experienced a much smaller bubble, peaking at 23.1% in Aichi in 1991.

**Table 6:** Estimated residential land price bubble sizes of Japan’s prefectures (%).

	Hokkaido	Aomori	Iwate	Miyagi	Akita	Yamagata	Fukushima	Ibaraki	Tochigi	Gumma	Saitama	Chiba	Tokyo	Kanagawa	Niigata	Toyama
**1980**	-	-	-	-1.5	-	-	-	-27.0	-18.8	-7.0	-23.5	-23.9	-28.4	-34.9	-	-15.3
**1981**	-	3.1	-	-	-	-	-	-19.0	-14.1	-	-6.8	-12.6	-20.6	-27.1	-	-
**1982**	-	12.3	-	-	-	-	-	-4.6	-5.6	-	-	-	-15.2	-16.1	-	-
**1983**	7.6	22.6	11.2	-	20.6	4.3	4.8	-	-	-	-	-	-9.1	-	1.3	-
**1984**	4.0	22.0	10.5	-	24.4	-	7.1	-	-	-	-	-	-8.6	-	2.5	-
**1985**	2.5	16.4	8.3	-	22.4	-	4.9	-	-	-	-	-	-7.1	-	-	-
**1986**	-	-	-	-	-	-	-	-	-	-	-1.3	-10.7	-	-	-	-
**1987**	-	-	-	-	-	-	-	-	-	-	-	-	80.4	12.2	-	-
**1988**	-	-	-	-	-	-	-	-	-	-	12.7	12.2	74.3	27.0	-	-
**1989**	-	-	-	-	-	-	-	-	-	-	17.3	27.3	59.8	21.9	-	-
**1990**	-	-	-	2.0	-	-	-	1.4	-	-	27.1	54.0	53.2	22.4	-	-
**1991**	-	-	-	3.8	-	-	-	8.7	8.4	6.1	22.7	45.7	42.0	17.7	-	-
**1992**	-	-	-	-	-	-	-	9.2	7.2	6.8	6.4	16.5	13.0	6.6	-	-
**1993**	-	-2.6	-	-	-	-	-	3.0	-	-	-	-	-	-	-	-
**1994**	-	-3.1	-	-	-	-	-	-	-	-	-	-	-	-	-	-
**1995**	-	-2.7	-	-	-	-	-	-	-	-	-	-	-1.4	-	-	-
**1996**	-	-2.5	-	-	-	-	-	-	-	-	-	-	-9.2	-	-	-
**1997**	-	-	-	-	-	-	-	-	-	-	-	-	-11.2	-	-	-
**1998**	-	-	-	-	-	-	-	-	-	-	-	-4.0	-14.1	-	-	-
**1999**	-	-	-	-	-	-	-	-	-	-	-3.9	-13.8	-20.2	-	-	-

**Table 7:** Estimated residential land price bubble sizes of Japan’s prefectures (%).

	Ishikawa	Fukui	Yamanashi	Nagano	Gifu	Shizuoka	Aichi	Mie	Shiga	Kyoto	Osaka	Hyogo	Nara	Wakayama	Tottori	Shimane
**1980**	-	-	-2.2	-	-9.5	-27.1	-25.9	-15.8	-22.4	-30.9	-32.9	-2.8	-25.2	-	-	-
**1981**	-	-	-	-	-3.7	-17.1	-15.1	-7.9	-15.5	-24.0	-24.1	-	-17.1	-	-	-
**1982**	-	-	-	-	-	-	-	-	-8.8	-	-16.3	-	-3.4	1.4	-	2.9
**1983**	-	-	-	7.8	-	-	-	-	-	-	-2.4	-	-	12.4	9.2	23.7
**1984**	-	-	-	9.3	-	-	-	-	-	-	-1.2	-	-	12.9	8.6	23.7
**1985**	-	-	-	6.7	-	-	-	-	-	-	-	-	-	11.7	9.4	18.0
**1986**	-	-	-8.3	-	-	-	-	-	-	-	-	-2.7	-2.5	-	-	-
**1987**	-	-	-9.1	-	-	-	-	-	-	-	-	-	-4.9	-	-	-
**1988**	-	-	-9.6	-2.0	-	-	-	-	-	-	-	-	-	-	-	-
**1989**	-	-	-	-	-	-	1.2	-	-	19.5	35.4	11.1	14.8	-	-	-
**1990**	-	-	-	-	-	13.5	22.5	-	38.4	85.6	95.4	44.1	73.0	-	-	-
**1991**	-	-	3.4	-	3.0	15.3	23.1	-	27.3	52.7	51.7	23.9	42.8	-	-	-
**1992**	-	-	4.5	-	5.1	9.9	9.0	-	12.1	4.8	12.1	-	16.5	-	-	-
**1993**	-	-	1.4	-	-	-	-	-	-	-	-	-	-	-	-	-
**1994**	-	-	-	-	-	-	-	-	-	-	-	-	-	-	-	-
**1995**	-	-	-	-	-	-	-	-	-	-	-	-	-	-	-	-
**1996**	-	-	-	-	-	-	-	-	-	-	-	-	-	-	-	-
**1997**	-	-	-	-	-	-	-	-	-	-	-	-	-	-	-	-
**1998**	-	-	-	-	-	-	-	-	-	-	-	-	-	-	-	-
**1999**	-	-	-	-	-	-	-	-	-	-	-	-4.0	-	-5.5	-	-

**Table 8:** Estimated residential land price bubble sizes of Japan’s prefectures (%).

	Okayama	Hiroshima	Yamaguchi	Tokushima	Kagawa	Ehime	Kochi	Fukuoka	Saga	Nagasaki	Kumamoto	Oita	Miyazaki	Kagoshima	Okinawa
**1980**	-6.3	-	-	-	-	-	-	-8.4	-	-	-	-	-	-	-23.2
**1981**	-	-	-	15.4	-	-	2.6	-	-	-	-	-	-	-	-2.6
**1982**	-	-	-	29.1	-	-	19.2	-	-	4.2	3.1	-	1.6	10.5	-
**1983**	-	10.7	-	30.5	5.9	5.9	43.2	-	10.7	11.7	20.2	17.9	15.1	22.6	-
**1984**	-	14.4	-	26.2	-	7.8	40.4	-	14.7	15.7	19.1	20.0	11.3	18.5	-
**1985**	-	16.1	-	22.2	-	4.6	35.9	-	13.0	12.9	16.3	20.2	9.4	16.0	-
**1986**	-	-	-	-4.1	-	-	-	-	-	-	-8.8	-	-	-	-3.0
**1987**	-	-	-	-7.8	-	-	-	-	-	-	-12.3	-	-	-	-
**1988**	-	-	-	-8.6	-	-	-	-	-	-	-11.8	-	-	-	-
**1989**	-	-	-	-5.9	-	-	-	-	-	-	-	-	-	-	-
**1990**	-	-	-	-3.9	-	-	-	-	-	-	-	-	-	-	-
**1991**	2.6	-	-	-	-	-	-	-	-	-	-	-	-	-	-
**1992**	-	-	-	-	-	-	-	-	-	-	-	-	-	-	-
**1993**	-	-	-	-	-	-	-	-	-	-	-	-	-	-	-
**1994**	-	-	-	-	-	-	-1.3	-	-	-	-	-	-	-	-
**1995**	-	-	-	-	-	-	-1.5	-	-	-	-	-	-	-	-
**1996**	-	-	-	-	-	-	-2.1	-	-	-	-	-	-	-	-
**1997**	-	-	-	-	-	-	-	-	-	-	-	-	-	-	-
**1998**	-	-	-	-	-	-	-	-	-	-	-	-	-	-	-
**1999**	-	-	-	-	-	-	-	-	-	-	-	-	-	-	-

Apparently, both commercial and residential land prices experienced serious bubbles in the 1980s Japan, but the bubble size of the former was much larger and more extensive than that of the latter. These results are coincident with the reality in the late 1980s Japan, suggesting that our panel data model can reliably estimate the real estate bubble size.

### 5.2. Estimation of bubble sizes in recent China

Based on the regression results of Models (5), (7) and (9), the bubble sizes of China’s 30 provinces/municipalities and 35 major cities are estimated according to Eq (8), as described in Tables 9, 10, 11, 12, 13 and 14, respectively.

**Table 9:** Estimated bubble sizes in China’s 30 provinces/municipalities (%).

	Beijing	Tianjin	Hebei	Shanxi	Inner Mongolia	Liaoning	Jilin	Heilongjiang	Shanghai	Jiangsu	Zhejiang	Anhui	Fujian	Jiangxi	Shandong
**1994**	10.9	1.7	-	0.2	22.7	-	29.0	25.0	-7.1	-	-11.5	-	9.2	-0.9	-
**1995**	-	-	-	-	29.3	-	-	0.2	-0.5	-	-13.3	-	-	-	1.2
**1996**	5.9	7.7	-	-	3.6	-	12.3	-	-	-	-14.3	-1.4	-	-	-
**1997**	26.5	-	2.9	-	7.2	-	-	-	-1.1	-	-6.1	-	-	-	-
**1998**	-	-	-	-	-	-	-	-	-	-	-3.5	-2.1	-	-8.6	-
**1999**	-	-	-	-	-	-	-	-	-5.2	-	-2.7	-	-	-3.9	-
**2000**	-	-	-	-	-	-	-	-	-9.0	-	-7.9	-	-	-	-
**2001**	-1.9	-	-	-	-	-	-	-	-6.6	-	-9.8	-7.2	-7.5	-	-
**2002**	-16.0	-0.4	-	-	-	-	-	-	-6.1	-	-4.4	-2.3	-6.7	-	-
**2003**	-24.0	-1-	-3.2	-	-	-	-	-	-	-	-2.9	-	-7.0	-	-
**2004**	-25.1	-	-6.1	-	-0.1	-	-	-	-	-	-1.6	-	-4.4	-	-
**2005**	-11.5	-	-2.3	-	-	-	-	-	-	-	-	-	-	-	-
**2006**	-1.8	-	-	-	-3.9	-	-0.4	-0.8	-	-	-	-	-	-	-
**2007**	-	-	-	-	-	-	-0.1	-	-	-	0.1	-	-	-	-
**2008**	-	-	-	-3.3	-6.3	-	-3.8	-	-	-	-	-	-	-	-
**2009**	-	-	-	-	-	-	-	-	17.5	-	19.2	-	-	-	-
**2010**	9.8	-	-	-	-	-	-	-	21.8	-	25.3	6.8	-	-	-
**2011**	-	-	-	-	-	-	-	-	14.7	-	20.9	6.1	5.1	12.7	-
**2012**	-	-	-	-	-	-	-	-	7.0	-	25.3	-	8.9	21.1	-
**2013**	-	-	-	-	-	-	-	-	19.0	-	23.1	-	6.8	24.1	-
**2014**	-	-	0.9	1.1	-	-	-	-	15.9	-	13.0	-	1.5	19.4	-

**Table 10:** Estimated bubble sizes in China’s 30 provinces/municipalities (%).

	Henan	Hubei	Hunan	Guangdong	Guangxi	Hainan	Chongqing	Sichuan	Guizhou	Yunnan	Shaanxi	Gansu	Qinghai	Ningxia	Xinjiang
**1994**	-	-	3.0	24.7	-	28.4	-	-8.8	-	5.8	20.9	-	-23.2	10.6	21.0
**1995**	-43.2	-	-	22.3	-	2.6	-	-	-	-	26.8	28.7	7.9	-	11.6
**1996**	-	-	-	25.1	-	-	-	-	-	-	-	0.8	-	-	15.1
**1997**	-	-	-	26.6	-	-	-	-	-	-	0.8	-	5.8	6.9	-
**1998**	-	0.6	-	6.2	-	-11.1	-	-	3.0	-	-	-	4.0	-	-
**1999**	-	-	-	-	-	-6.9	-	-	-	-	-5.0	-	14.0	0.5	-
**2000**	-	-	-	-	-	-3.7	-	-	-	-	-	-	-	-	-
**2001**	-	-	-	-	6.0	-12.5	-	-	-	1.3	-	-	-	-	-
**2002**	-	-	-	-	4.3	-23.3	-	-	-	-	-	-	-	6.9	-
**2003**	-	-2.3	-	-9.6	-	-15.5	-	-2.5	-	-	-	-3.9	-	-	-
**2004**	-	-	-	-4.5	-	-14.9	-	-2.4	-	-	-	-	-	-	-
**2005**	-	-	-	-	-	-	-	-	-	-	-	-	-	-	-1.9
**2006**	-	-	-	-0.8	-	-	-	-	-	-	-	-1.2	-	-	-8.2
**2007**	-	-	-	-	-	-	-	-	-	-	-	-	-	-5.1	-6.1
**2008**	-	-	-	-0.7	-	1.0	-	-	-	-	-	-11.6	-	-8.7	-9.3
**2009**	-	-	-	-	-	9.4	-	-	-	-	-	-	-	-	-
**2010**	-	-	-	-	-	32.8	-	0.4	-	-	-	-	-	-	-
**2011**	-	-	-	-	-	20.0	-	5.2	-	-	-	-	-	-1.4	-
**2012**	-	-	-	-	-	-	-	7.5	-	-	-	-	-	-2.5	-
**2013**	0.4	-	-	-	-	0.6	-	1.9	-	-	-	-	-	-1.7	-
**2014**	-	-	-	-	-	1.6	-	-	-	-	-	-	-	-8.2	-

**Table 11:** Estimated bubble sizes of commercial commodity buildings in China’s major 35 cities (%).

	Beijing	Tianjin	Shijiazhuang	Taiyuan	Huhehaote	Shenyang	Changchun	Haerbin	Shanghai	Nanjing	Hangzhou	Hefei	Fuzhou	Nanchang	Jinan	Zhengzhou	Wuhan	Changsha
**2001**	-	-	-	-7.2	24.4	-	-	-	-	-	-	-	-23.1	-11.1	-12.7	-	-7.4	-
**2002**	-	-	10.1	-	9.9	-	15.9	-	-	-1.5	-	-	-	-	-40.2	0.2	-38.4	-5.4
**2003**	-	-	-22.5	-	-	-	-	-	-	-	-	-	-22.9	-	-13.2	-	-14.6	-
**2004**	-9.1	-	-38.8	-	-	-	-	-	-	-	-	-	-11.0	-7.9	1.6	-	-	-
**2005**	-	-2.9	-	-	19.2	-	-	-	-	-	-	-	-	-	-	-	-	-
**2006**	-	-	-	-	-	-	-	-	-12.2	-	-	-	2.8	-	-	-	12.5	-
**2007**	-	-	-	-	-	-	-	-	-19.6	-	-	-	-	-	-9.4	-	1.9	-
**2008**	-	-	-44.5	-7.8	-	-	-	-	-25.6	-	-	-	-	3.0	-	-	-	-7.1
**2009**	-	-	27.7	-	-	-	-	-	4.5	-	-	-	-	-	11.9	-	5.6	-
**2010**	-	-	14.8	-	-	-	-	-	-	-	-	-	-	-	24.7	-	-	-
**2011**	-	-	13.1	-6.4	-	-	-	1.8	14.2	-	-	-	10.7	1.7	-	-	4.2	-
**2012**	-	-	-	23.7	-	-	-	-	-	-	-	-	-	-	2.5	-	-	-
**2013**	-	-	8.3	3.8	-	-	-	-	3.8	-	-	-5.4	28.2	3.7	-	-	-	-
**2014**	-	-	-	3.4	-10.4	-	-	-	12.6	-	-	-19.4	-	-	-	-0.1	-	-

**Table 12:** Estimated bubble sizes of commercial commodity buildings in China’s major 35 cities (%).

	Nanning	Haikou	Chongqing	Chengdu	Guiyang	Kunming	Xiaan	Lanzhou	Xining	Yinchuan	Wulumuqi	Dalian	Qingdao	Ningbo	Xiamen	Shenzhen	Guangzhou
**2001**	14.5	-	-	-	-2.7	-	-	-	-	47.8	33.7	-	-	-6.2	-13.8	-	-
**2002**	2-	37.8	-	-	-	3.8	-15.5	-	7.7	25.3	48.7	-	-1.7	-	-	1.6	-
**2003**	-	30.1	-	-	-	-	-	2.8	-	22.3	36.5	-	-	-	-5.6	-	-
**2004**	16.2	-33.2	-	-	-11.4	-3.3	-	-	-	36.9	-	-	-	-	-	-	-
**2005**	-	-	-	-	5.0	-	6.0	-	-	22.2	35.9	-	-	-	4.2	-	-
**2006**	-	-	-	-	-	2.2	13.5	-	-12.2	-	-12.8	-	-	-	-	-	-
**2007**	-11.3	-	-	-	1.7	-	-	-13.5	-	-3.5	-11.7	1.4	-	-	-	-	-3.5
**2008**	-	-	-	-	7.2	-	-	-	-	-0.4	-19.2	-	-	-	-	-25.7	-
**2009**	-3.6	-	-	-	-	-	-	-3.1	-1.5	-	-10.7	-	-	-	-	-	-
**2010**	-	-	-	-	-	-	-	-	-1.6	-18.5	-13.9	-	-	-	-	-	-
**2011**	-11.7	-	-	-	-	-	-	-	-	-17.4	-	-	-	-	-	-	-
**2012**	-	35.9	-	0.5	-	-	-	-	-	-5.2	-	-	10.1	-	-	-	-
**2013**	7.6	-	-	-	-	-5.2	-	-	25.9	-8.3	-	-	2.3	-	-	-	-
**2014**	-	-	-	-	-3.9	-	-	9.4	-	-9.7	-	-	-	-	-	-	-

**Table 13:** Estimated bubble sizes of residential commodity buildings in China’s major 35 cities (%).

	Beijing	Tianjin	Shijiazhuang	Taiyuan	Huhehaote	Shenyang	Changchun	Haerbin	Shanghai	Nanjing	Hangzhou	Hefei	Fuzhou	Nanchang	Jinan	Zhengzhou	Wuhan	Changsha
**2001**	-	-	8.2	-	46.1	15.8	4.2	-	-9.0	-	-15.9	6.2	-16.4	-	-	8.0	-	18.4
**2002**	-1.6	-	-	-	-	5.6	-	-	-7.1	-	-6.6	2.9	-22.7	-	-	1.2	-	3.9
**2003**	-11.9	-	-	-	-	1.3	-	-	-	-	-5.2	6.9	-22.9	-	-	-	-	2.1
**2004**	-14.1	-	-11.8	-	-	-	-	-	-	-	-11.7	11.5	-21.2	-	-	-	-	-
**2005**	-4.2	-	-3.6	-	-7.5	-	-	-	-	-	-	-	-1.2	-	-	-	-	-
**2006**	-	-	-	-	-	-	-	-0.2	-	-	-	-	-	-	-	-	-	-
**2007**	-	2.4	-	-	-	-	-	-	-	-	-	-	-	-	-	-	6.7	-
**2008**	-	-	-	-4.3	-0.4	-	-	-	-1.8	-3.7	-	-	-	-	-	-	-	-
**2009**	0.8	-	-	-	-	-	-	-	4.6	-	6.2	-	3.7	-	-	-	-	-
**2010**	17.2	-	-	15.6	-	-	1.3	1.2	9.8	9.3	27.4	-	11.2	-	-	-	-	-
**2011**	-	-	-	-	-	-	3.8	-	-	-	1.0	-	18.4	-	-	-0.1	-	-
**2012**	-	-	-	-	-	-	-	-	-	-	-	-6.8	20.9	-	-	-	-	-
**2013**	-	-	-	-	-	-	-	-	4.6	-	2.6	-9.4	6.9	-	-	-	-	-
**2014**	-	-0.2	1.7	-	-	-0.1	-	-	0.2	-	-	-4.1	-	-	-	-	-	-9.4

**Table 14:** Estimated bubble sizes of residential commodity buildings in China’s major 35 cities (%).

	Nanning	Haikou	Chongqing	Chengdu	Guiyang	Kunming	Xiaan	Lanzhou	Xining	Yinchuan	Wulumuqi	Dalian	Qingdao	Ningbo	Xiamen	Shenzhen	Guangzhou
**2001**	4.1	-	-	-1.2	-	9.4	-	-	10.4	38.3	8.4	-	-	-24.8	-17.0	-	-
**2002**	2.3	-	-	-1.8	-	-	-	-1.2	2.1	42.4	2.7	-	-	-13.7	-24.5	-2.2	-
**2003**	-	-8.6	-	-2.9	-	-	-	-	3.3	15.9	-	-	-	-18.1	-20.2	-6.2	-
**2004**	-	-5.4	-	-	-	-	-	-	-	12.6	-	-	-	-14.9	-12.4	-9.1	-
**2005**	-	-4.4	-	-	-	-	-	-	-	-	-	-	-	-	-	-10.3	-
**2006**	-	-9.8	-	-	-	-	-	-	-	-	-	-	-	-	-	-	-
**2007**	-	-	-	3.3	-	-	-	-	-	-	-	-	-	-	9.5	-	-
**2008**	-	-	-	4.6	-	-	-	-	-	0.1	-	-	-	-	0.9	-	-
**2009**	-	4.0	-	-	-	-	-	-	-	-	-	-	-	12.8	-	-	-
**2010**	-	36.0	-	-	4.7	-5.3	-	-	-	-	-	-	-	27.8	6.9	10.4	-
**2011**	-	-	-	-	-	-	-	-	-4.0	-4.3	-	-	-	10.0	6.4	7.3	-
**2012**	-	-	-	-	-	-	-	-	-	-9.9	-	-	-	4.5	-	-	-
**2013**	-	-	-	-	-3.6	-	-	-	-	-10.4	-	-	-	-	1.7	1.0	-
**2014**	-1.7	-	-	-	-7.5	-	-0.1	-	-	-22.8	-	-	-	-	17.2	-	-

Table 5 shows that the average commodity building prices of 11 provinces/municipalities moderately overpriced in recent years are clustered in five areas. The largest housing overpricing area was Hainan, peaking at 32.8% in 2010. The second one was the Eastern Coast Area, where housing prices in Zhejiang and Shanghai were overvalued by 25.3% and 21.8%, respectively. The Southeast Area was third, where houses in Jiangxi and Fujian were overpriced at 24.1% and 8.9% in 2013 and 2012, respectively, while the Northern area was fourth with Beijing, Shanxi and Hebei peaking at 9.8%, 1.1% and 0.9% in 2010, 2014 and 2014, respectively. Housing prices of the Southwestern Area were slightly overvalued, peaking at 7.5% in Sichuan in 2012.

According to Tables 11, 12 and 15 cities experienced overvalued commercial commodity building prices from 2007 to 2014. These cities are geographically clustered in four main areas. The most overpriced was Hainan Island whose capital Haikou had a bubble of 35.9% in 2012. The second largest was the Coast Area, where Fuzhou had the highest overvaluing of 28.2% in 2013, followed by Shanghai at 14.2% in 2011 and Qingdao with 10.1% in 2012. The third one was the Northern Area, where overpricing in Shijiazhuang, Jinan and Taiyuan peaked at 27.7%, 24.7% and 23.7% in 2009, 2010 and 2012, respectively. Xining in the Northwest Area peaked at 25.9% in 2013 and Lanzhou at 9.4% in 2014. Followed by Nanning, Guiyang and Chengdu in the Southwest Area, Nanchang and Wuhan in the Middle Area, and Haerbin in the Northeast Area with slight overpricing.

**Table 15:** Ratios of the average apartment price to the average monthly salary per capita in Tokyo and Osaka in Japan*.

Year	1975	1976	1977	1978	1979	1980	1981	1982	1983	1984	1985	1986	1987
**Tokyo**	1.7	1.4	1.4	1.3	1.4	1.5	1.5	1.5	1.4	1.3	1.3	1.3	1.9
**Osaka**	1.0	0.9	0.9	0.9	0.9	1.0	1.0	1.0	1.0	0.9	0.9	0.9	0.9

*Source: Statistics Bureau of Japan—the average apartment price (yen/sq. m.), and the average monthly earnings of regular employees by prefectures (Establishments with 30 or more Employees).

Tables 13 and 14 describes that the average residential commodity building prices of 17 cities were moderately overpriced from 2007–2014, clustered mainly in four areas. Haikou had a highest bubble size of 33.6% in 2010. The second largest was the Eastern Coast Area, where Ningbo and Hangzhou had the highest overvaluing of 27.8% and 27.4% in 2010, respectively, followed by Shanghai with 9.8% and Nanjing at 9.3% in 2010. Fuzhou in the Southeast Coast Area peaked at 20.9% in 2012 and Xiamen at 17.2% in 2014, while Shenzhen peaked at 10.4% in 2010. The fourth one was the Northern Area, where overpricing in Beijing peaked at 17.2% in 2010, followed by Taiyuan with 15.6% in 2010, Tianjin at 2.4% in 2007 and Shijiazhuang with 1.7% in 2014. Additionally, Wuhan in the Middle Area, Chengdu and Guiyang in the Southwestern Area, and Changchun and Haerbin in the Northeastern Area were slightly overpriced.

Consequently, the analyses at both provincial and city levels show that only a few regions in China had moderate overpricing in commercial housing, mainly in Hainan Island, the Eastern Coast Area, and the Southeast Area. Hence, the issue of the housing price bubble is not as serious as the public’s concerns suggest.

### 5.3. Comparison of the bubbles in Japan and China

**Fig 1:**
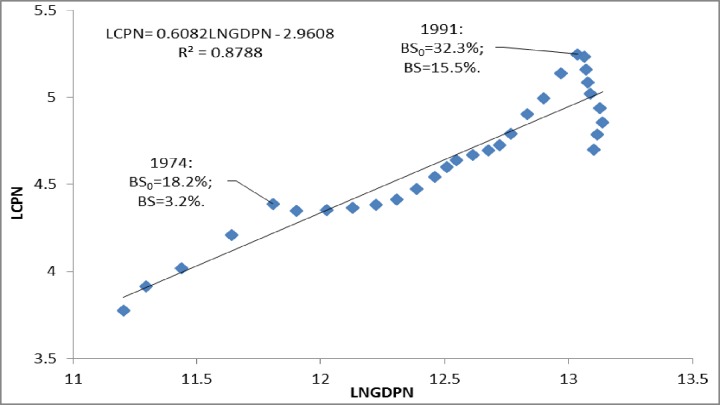
Regression results of LCPN to LNGDPN in Japan from 1970 to 1999*. *Source: Statistics Bureau of Japan—all urban commercial land price index, and gross domestic product at current prices. Notes: Bubble size is calculated according to Eq (8). BS_0_ does not remove rational fluctuations (e_it_), while BS removes rational fluctuations (e_it_).

Based on the above analysis, real estate overpricing in China in recent years is much smaller than that in Japan in the late 1980s, which is contrary to the recent perception. To have a better understanding, we also compared the long time series data in Japan at the national level with those in China at both the national level and the 35 major cities level. Fig 1 shows the regression of the logarithmic national commercial land price index (LCPN) to nominal economic growth (LNGDPN) in Japan from 1970 to 1999. The commercial land price index peaked in 1974 for the first time. After a downward fluctuation in 1975, the land prices began rising again. The increase accelerated from 1987, and peaked for the second time in 1991. Thereafter, the bubble burst. The first time in 1974, overpricing peaked at 3.2%, but peaked at 15.5% the second time in 1991. Overpricing in Japan in 1974 disappeared slowly with the economic growth rather than the sudden bubble burst like in 1991.

**Fig 2:**
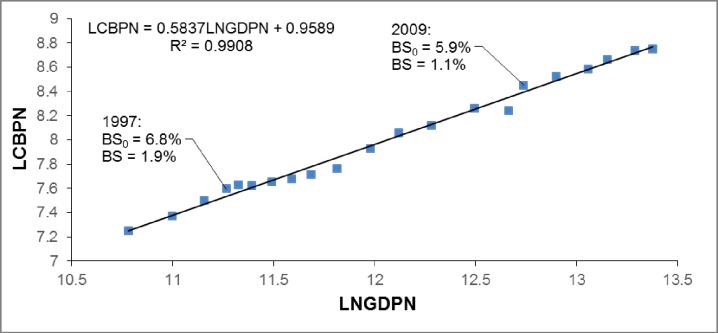
Regression results of LCBP35 to LNGDP35 in China’s major 35 cities from 1999 to 2014*. *Source: CEIC—commercial housing prices of 35 major cities, and town city gross domestic product at current prices. Data were used to calculate the sum of the 35 major cities. Notes: Bubble size is calculated according to Eq (8). BS_0_ does not remove rational fluctuations (e_it_), while BS removes rational fluctuations (e_it_).

**Fig 3:**
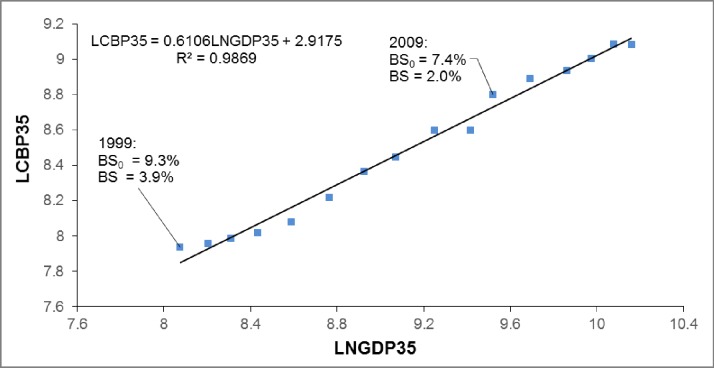
Regression results of LCBPN to LNGDPN in China from 1994 to 2014*. *Source: China Statistical Yearbook (various years)- national commercial housing price, and national gross domestic product at current prices. Notes: Bubble size is calculated according to Eq (8). BS_0_ does not remove rational fluctuations (e_it_), while BS removes rational fluctuations (e_it_).

Figs 2 and 3 illustrate the regression of logarithmic average commodity building prices to economic growth in China for the 35 major cities level (LCBP35 to LNGDP35) from 1999 to 2014 and the national level (LCBPN to LNGDPN) from 1994 to 2014, respectively. The average commodity building price in the 35 major cities was overpriced at 3.9% in 1999 but only 2.0% in 2009. Fig 3 suggests that the national average commodity building price was overvalued at 1.9% in 1997 but decreased to 1.1% in 2009. Compared with Japan, these results imply that the overpricing of real estate in recent China is much smaller than the real estate bubble in 1980s Japan, but is similar to the moderate overvaluing in 1970s Japan. Thus, the housing price over-increase in recent China is not as serious as the land price bubble in 1980s Japan, and may result in a soft landing similar to that in 1970s Japan.

Some might argue that the big difference between the housing price and people’s income is evidence of an extreme bubble. Thus, we also calculated the ratio of apartment price (yen/sq. m.) to monthly average salary per capita in Tokyo and Osaka in Japan with that for the commodity building price (yuan/sq. m.) in Beijing and Shanghai in China.

Tables 15 and 16 illustrates that from 1975 to 1999 the ratio in Tokyo and Osaka mostly fluctuated around 1.3–1.7 and 0.9–1.1, respectively, but increased sharply from 1987 and peaked at 3.0 in 1990 and 2.2 in 1991, respectively, due to the land price bubble bursting. Table 17 shows that the ratio in Beijing and Shanghai fluctuated from 2.2 to 3.2 and from 1.8 to 2.6, respectively, in 2001–2014. There is no obvious upward trend in these two Chinese cities, suggesting that there is no sign of a housing price bubble based on the income–price perspective. Because only the husband works at a formal long-term job in most Japanese families, while both spouses work in China, it is reasonable that the absolute values of the ratios in Chinese cities are higher than those in Japanese cities.

**Table 16:** Ratios of the average apartment price to the average monthly salary per capita in Tokyo and Osaka in Japan*.

Year	1988	1989	1990	1991	1992	1993	1994	1995	1996	1997	1998	1999
**Tokyo**	2.5	2.9	3	2.9	2.3	1.8	1.7	1.4	1.4	1.3	1.3	1.4
**Osaka**	1.2	1.6	2.1	2.2	1.6	1.4	1.3	1.2	1.1	1.1	1.1	1.1

*Source: Statistics Bureau of Japan—the average apartment price (yen/sq. m.), and the average monthly earnings of regular employees by prefectures (Establishments with 30 or more Employees).

**Table 17:** Ratios of the average commodity building price to the average monthly salary per capita from 2001 to 2014 in Beijing and Shanghai in China*.

	2001	2002	2003	2004	2005	2006	2007	2008	2009	2010	2011	2012	2013	2014
**Beijing**	3.2	2.6	2.2	2.2	2.4	2.6	3.0	2.6	2.8	3.2	2.6	2.4	2.4	2.2
**Shanghai**	2.2	2.0	2.2	2.6	2.4	2.2	2.2	1.8	2.4	2.4	2.2	2.2	2.2	2.0

*Source: China Statistical Yearbook (various years) - the average yearly salary per capita, and the average commercial housing price.

Consequently, although China had a small overpricing in housing prices in a few areas, it is not as serious as a real estate bubble. This moderate overvaluing should be absorbed by the economic growth rather than a sudden bubble burst if there is a stable economic development. However, since Chinese cities are much wider than Japanese prefectures, the average housing prices that we adopted are diluted by the large suburban districts of Chinese cities. For example, the area of Beijing is five times that of Tokyo, and the suburban area is much more expansive than the downtown area. Thus, the housing price in the downtown area is much higher than the average housing price we employed, implying that the actual bubble size in the central city would be much higher than we estimated. Besides, the latest data available in our models are before 2014, which are not able to indicate the situation after 2014. Although the overheated real estate market tended to be cooled in 2014, slowing down the sharp growth in housing price from 1999–2013, housing prices soared again in 2016 in major cities, such as Bejing, shanghai, Nanjing and Hangzhou. The new round of rising in housing prices may expand the existing overpricing before 2014 to a risky bubble, which should be taken seriously. Accordingly, China should keep the high economic growth and gradually reduce the increase of housing prices in the central metropolis.

## 6. Conclusions

Based on the theoretical equilibrium price of real estate determined by local economic fundamentals, we established a measurement model for real estate bubble size using the methodology of panel data analysis. The reliability of our model on real estate bubble size measurement is evidenced by its application in Japan with the panel data of 47 Japanese prefectures from 1980 to 1999. Then, employing 30 Chinese provinces/municipalities from 1994 to 2014 and 35 major Chinese cities from 2001 to 2014, the model estimated the real estate bubble sizes in different areas. This study reveals the followings.

First, there was a commercial land price bubble in Japan. The Kanto, Kinki, and Chubu areas experienced serious bubbles, while the Kyushu and Tohoku areas had medium-sized bubbles. Chugoku and Hokkaido had small bubbles, and the Shikoku area had a slight bubble. The bubbles of Tokyo, Osaka, and Kyoto peaked at 98.5% in 1987, 133.2% in 1990, and 94.7% in 1990, respectively.

Second, 18 Japanese prefectures experienced obvious residential land price bubbles in the late 1980s. These prefectures were geographically clustered in three areas: Tokyo-centralized, Osaka-centralized, and Aichi-centralized areas. The former two regions experienced much larger bubbles. The bubble peaked at 80.4% in Tokyo in 1987, at 95.4% in Osaka in 1990, and at 23.1% in Aichi in 1991. Accordingly, the commercial and residential land prices had serious bubbles in the 1980s, which coincides with reality in 1980s Japan, suggesting that our model is reliable. This would be the first study to use the Japanese case in the 1980s to test the reliability of the model to estimate the bubble size.

Third, 10 of the 30 provinces/municipalities in China experienced commodity building overpricing from 2007 to 2014, while commercial and residential commodity building prices overpriced in 15 and 17 of the 35 major cities, respectively. But they were clustered mainly in four regions: (1) the Hainan island region where the both commercial and residential building prices of Haikou were overvalued at 36.0% in 2010, (2) the Eastern Coast Area where overpricing of residential building prices peaked at 27.8% in Ningbo, 27.4% in Hangzhou, 9.8% in Shanghai and 9.3% in Nanjing in 2010, (3) the Southeast Area, centralized by Fuzhou, Xiamen and Shenzhen, which were overvalued at 20.9%, 17.2%, 10.4% for residential building prices, respectively, (4) the Northern Area, centralized by Beijing, overpricing of residential building prices peaked at 17.2% in 2010. In addition, a slight overvaluing appeared in the Southwestern Area and Northeastern Area.

Fourth, the housing price over-increase in China in recent years is much smaller than that in Japan in the late 1980s, and similar to the moderate overpricing in 1970s Japan. Another contribution of this study is that it compares the bubble sizes between the two countries. Although the overpricing of Chinese housing appeared in a few regions, it is not as serious as that in 1980s Japan. To increase the odds of a soft landing of Chinese real estate prices similar to Japan in the 1970s, China should maintain high economic growth while taking reasonable measures to slowly decrease housing prices in central metropolises.

## Supporting information

S1 File Details of Data Sources. (DOCX)
