# Supplementary material for: A measurement model for real estate bubble size based on the panel data analysis: An empirical case study
Source: PLoS One. 2017 Mar 8;12(3):e0173287. doi: 10.1371/journal.pone.0173287 (PMC5342231; doi:10.1371/journal.pone.0173287)
Supplement: S1 File — (DOCX) [file pone.0173287.s001.docx]

**Details of Data Sources**

**1. Data of Japan**

(1) The average commercial land price (CP) by prefecture:

Japan Statistics Bureau, Historical Statistics of Japan, Contents, Chapter 15 Real Estate and Land, Price and Rent, 15-20 Average Prices of Housing Land by Use and Prefecture (Per 1sq. m) (1980--2004)(Excel:140KB), Commercial Site, Year: 1980-1999

URL: http://www.stat.go.jp/english/data/chouki/15.htm

(2) The average residential land price (RP) by prefecture:

Japan Statistics Bureau, Historical Statistics of Japan, Contents, Chapter 15 Real Estate and Land, Price and Rent, 15-20 Average Prices of Housing Land by Use and Prefecture (Per 1sq. m) (1980--2004)(Excel:140KB), Residential Site, Year: 1980-1999

URL: http://www.stat.go.jp/english/data/chouki/15.htm

(3) Nominal GDP by prefecture:

Japan Statistics Bureau, Historical Statistics of Japan, Contents, Chapter 3 National Accounts, Prefectural Accounts, 3-37- a Prefectural Accounts - 68SNA, Benchmark year = 1990 (F.Y.1975--1999)(Excel:54KB), Gross prefectural domestic product (at current price), Year: 1980-1999

URL: http://www.stat.go.jp/english/data/chouki/03.htm

(4) Population by prefecture:

Japan Statistics Bureau, Historical Statistics of Japan, Contents, Chapter 2 Population and Households, 2- 5 Population by Prefecture and Sex (1884--2009)(Excel:139KB), Year: 1980-1999

URL: http://www.stat.go.jp/english/data/chouki/02.htm

(5) Administer area by prefecture:

Japan Statistics Bureau, Historical Statistics of Japan, Contents, Chapter 1 Land and Climate, 1-11 Area by Prefectures (1920--2010) (Excel:42KB), Year: 1980-1999

URL: http://www.stat.go.jp/english/data/chouki/01.htm

(6) The lending rate (LI):

Japan Statistics Bureau, Historical Statistics of Japan, Contents, Chapter 14 Finance and Insurance, Interest Rate, 14- 1 Principal Interest Rates (1946--2004)(Excel:63KB), Lending rate, Prime Lending Rate, Short-term, Year: 1980-1999

URL: http://www.stat.go.jp/english/data/chouki/14.htm

(7) Commercial land price index in national level (LCPN):

Japan Statistics Bureau, Historical Statistics of Japan, Contents, Chapter 22 Prices, Price of Land, 22-20 Urban Land Price Index - All Urban Land, 6 Major Cities and excluding 6 Large Cities (1955--2005)(Excel:32KB), Year: 1980-1999

URL: http://www.stat.go.jp/english/data/chouki/22.htm

(8) Nominal GDP in national level (GDPN):

Japan Statistics Bureau, Historical Statistics of Japan, Contents, Chapter 3 National Accounts, [68SNA] Flow, 3- 3- a Gross Domestic Product Classified by Economic Activities (Major Industry Group) (At Current Prices, At Constant Prices, Deflators) - 68SNA, Benchmark year = 1990 (1955--1998)(Excel:61KB), Gross domestic product (not including statistical discrepancy), Year: 1970-1999

URL: <http://www.stat.go.jp/english/data/chouki/03.htm>

**2. Data of China:**

(1) The average commodity building prices (CBP) by province:

National Bureau of Statistics of China, National Data, regional, Annual by Province, Indicators, Investments in Fixed Assets and Real Estate, Average Selling Price of Commercialized Buildings by use, Region: Beijing/Tianjin/..., Average Selling Price of Commercialized Buildings(yuan/sq. m), Year: 1994-2014

URL: http://data.stats.gov.cn/english/easyquery.htm?cn=E0103

(2) Nominal GDP by province:

National Bureau of Statistics of China, National Data, regional, Annual by Province, Indicators, National Accounts, Gross Regional Product, Region: Beijing/Tianjin/..., Gross Regional Product(100 million yuan), Year: 1994-2014

URL: http://data.stats.gov.cn/english/easyquery.htm?cn=E0103

(3) Population by provinces:

National Bureau of Statistics of China, National Data, regional, Annual by Province, Indicators, Population, Region: Beijing/Tianjin/..., Resident Population (year-end) (10000 persons), Year: 1994-2014

URL: http://data.stats.gov.cn/english/easyquery.htm?cn=E0103

(4) Administer area by province:

CEIC Database, China, Annual, Territorial Resources, Administer Area, by Province, Region: Beijing/Tianjin/..., Year: 1994-2014

(5)The average residential commodity building price (RCP) by city:

National Bureau of Statistics of China, National Data, regional, Annual by Selected Cities, Indicators, Real Estate, Region: Beijing/Tianjin/..., Average Selling Price of Commercialized Residential Buildings (yuan/sq. m), Year: 2001-2014

URL: http://data.stats.gov.cn/english/easyquery.htm?cn=E0105

(6)The average commercial commodity building price (CCP) by city:

CEIC Database, China, Annual, Real Estate, Average Selling Price of Commercialized Buildings by use, 35 cities, Average Selling Price of Houses for Business Use(yuan/sq. m), Region: Beijing/Tianjin/... , Year: 2001-2014

(7)Population by city:

National Bureau of Statistics of China, National Data, regional, Annual by Selected Cities, Indicators, Population and Employment, Region: Beijing/Tianjin/..., Total Population (year-end) (10000 persons), Year: 2001-2014

URL: http://data.stats.gov.cn/english/easyquery.htm?cn=E0105

(8)Nominal GDP by city:

National Bureau of Statistics of China, National Data, regional, Annual by Selected Cities, Indicators, National Accounts, Region: Beijing/Tianjin/..., Gross Domestic Product (100 million yuan), Year: 2001-2014

URL: http://data.stats.gov.cn/english/easyquery.htm?cn=E0105

(9)Administer area by city:

CEIC Database, China, Territorial Resources, Administer Area, by cities, Region: Beijing/Tianjin/..., Year: 2001-2014

(10)The lending rate (LI):

CEIC Database, China, Finance, interest rate, lending rate, short-term lending rate: 6 months-1 year (including 1 year), Year: 1994-2014

(11)The average commodity building price in 35 major cities (CBP35):

CEIC Database, China, Annual, Real Estate, Average Selling Price of Commercialized Buildings by use, 35 cities, Average Selling Price of Commercialized Buildings(yuan/sq. m), Region: 35 cities , Year: 2001-2014
